# Iron Oxide Nanoparticle-Mediated mRNA Delivery to Hard-to-Transfect Cancer Cells

**DOI:** 10.3390/pharmaceutics15071946

**Published:** 2023-07-14

**Authors:** Jianxi Huang, Guanyou Lin, Taylor Juenke, Seokhwan Chung, Nicholas Lai, Tianxin Zhang, Tianyi Zhang, Miqin Zhang

**Affiliations:** 1 Department of Materials Science and Engineering, University of Washington, Seattle, WA 98195, USA; 2 Department of Chemical Engineering, University of Washington, Seattle, WA 98195, USA; 3 Department of Biology, University of Washington, Seattle, WA 98195, USA

**Keywords:** iron oxide nanoparticle, layer-by-layer, mRNA delivery, mRNA therapy, cancer

## Abstract

mRNA-based therapeutics have emerged as a promising strategy for cancer treatment. However, the effective delivery of mRNA into hard-to-transfect cancer cells remains a significant challenge. This study introduces a novel approach that utilizes iron oxide nanoparticles (NPs) synthesized through a layer-by-layer (LbL) method for safe and efficient mRNA delivery. The developed NPs consist of an iron oxide core modified with a thin charge-bearing layer, an mRNA middle layer, and an outer layer composed of perfluorinated polyethyleneimine with heparin (PPH), which facilitates efficient mRNA delivery. Through a comparative analysis of four nanoparticle delivery formulations, we investigated the effects of the iron oxide core’s surface chemistry and surface charge on mRNA complexation, cellular uptake, and mRNA release. We identified an optimal and effective mRNA delivery platform, namely, (IOCCP)-mRNA-PPH, capable of transporting mRNA into various hard-to-transfect cancer cell lines in vitro. The (IOCCP)-mRNA-PPH formulation demonstrated significant enhancements in cellular internalization of mRNA, facilitated endosomal escape, enabled easy mRNA release, and exhibited minimal cytotoxicity. These findings suggest that (IOCCP)-mRNA-PPH holds great promise as a solution for mRNA therapy against hard-to-transfect cancers.

## 1. Introduction

As a major public health threat, cancer is one of the leading causes of death with a projected death toll of 1,958,310 in the United States in 2023 [1]. Despite advances made in standard treatments using chemotherapy and radiotherapy, treatment outcomes for certain forms of cancer remain devastatingly ineffective [2,3,4]. For example, some forms of breast and brain cancer cells have been shown to develop resistance to withstand chemotherapy and radiotherapy [5,6,7]. Gene delivery has emerged as a promising strategy for cancer treatment due to its capability of regulating therapeutic or pathogenic genes within tumors. Specifically, mRNA delivery has garnered immense interests in vaccination and immunotherapy applications due to its rapid cellular expression of therapeutic genes. Unlike DNA delivery, mRNA delivery does not require host genome integration or nuclear entry for transfection, which significantly alleviates the safety concern of insertional mutagenesis. However, the delivery of mRNA is not without challenges. A carrier is usually required to deliver mRNA due to the large size, high immunogenicity, structural instability of mRNA, as well as the difficulty of mRNA to be internalized by cells [8]. Since the single-stranded mRNA is more chemically unstable than the double-stranded DNA and physically larger than siRNA, the requirement for an mRNA nanocarrier in terms of cargo protection and release is more stringent than its counterpart designed to deliver DNA or other small RNAs [9,10]. To address these issues, a myriad of viral and non-viral nanocarriers have been investigated that can load, protect and transport mRNA.

The use of viral carriers for mRNA delivery is effective but limited by its immunogenicity, cytotoxicity, and small payload size [8]. Non-viral carriers, on the other hand, provide versatility in terms of physicochemical modification and functional designs, offering the ability to tailor them as a biocompatible and stable delivery system with high loading capacity. Among non-viral carriers, nanoparticles with inorganic cores have shown promise for mRNA delivery. This can be attributed to their small sizes, which allow for stable circulation in the body and effective uptake by cancer cells, as well as their large surface area-to-volume ratio, enabling efficient loading of mRNA [11]. Superparamagnetic iron oxide nanoparticles (SPION) have been extensively studied as nanocarriers for delivering therapeutic nucleic acids, including DNA and siRNA [12,13,14]. SPIONs have also been widely used in various biological applications due to their desirable properties such as biocompatibility and biodegradability [15]. Furthermore, their superparamagnetic nature makes them well-suited as contrast agents for magnetic resonance imaging (MRI). This characteristic enables non-invasive and real-time visualization of gene delivery at tumor sites [12,13,14].

A typical SPION nanocarrier structure comprises an iron oxide core, a middle layer of highly cationic coatings, and an outer layer of nucleic acid [16]. Various cationic coatings such as polyethyleneimine (PEI), poly(l-lysine), poly(amido amine), polyesters, and chitosan have been investigated to promote gene protection, cellular internalization, and endosomal escape [10]. In these designs, the highly cationic moieties in the polymer coatings are critical for binding and condensing the anionic nucleic acids via electrostatic interaction and protect them from nucleases. However, an excessively strong electrostatic attraction between these components can impede the subsequent release of the nucleic acids in the cytoplasm [17]. Therefore, the conventional design of SPION with highly cationic polymer coatings faces challenges in achieving effective binding during nanocarrier transportation and facilitating the smart release of free mRNA upon cellular internalization.

The layer-by-layer (LbL) technique offers an alternative approach for loading genetic materials onto nanoparticles. In this technique, anionic genetic materials are deposited onto a weak-cationic core nanoparticle, followed by coating the particle with an outer layer of weak-cationic polymer. The weak-cationic core serves as a sturdy support for gene loading, allowing for precise control of the size and improved uniformity of nanoparticles. The outer weak-cationic polymer layer enhances condensation capability and provides physical protection for the genetic materials situated in the middle layer. This LbL approach enables effective gene loading, improved condensation, and enhanced protection within the nanoparticle structure. With genetic material such as mRNA sandwiched between the weak-cationic core and the weak-cationic outer layer, the condensation and release of mRNA on nanoparticles can be balanced for effective mRNA delivery into cancer cells. In our initial investigation, we identified a potential candidate for the weakly cationic outer layer—a perfluorinated, low molecular weight PEI with heparin (PPH). Low molecular weight PEI possesses weak charges and abundant amine groups for functional modification. The addition of fluorination groups enhances cellular uptake by enabling the increased penetration of the cell membranes, assisted by their amphiphobic properties. Furthermore, the heparin modification facilitates interaction with fibroblast growth factor receptors, leading to further improved cellular uptake [18]. The PPH construction has the advantages of facilitating mRNA delivery to the cellular membrane and promoting intracellular release of mRNA (Figure S1). In this study, we report the development of an LbL nanoparticle composed of an SPION core, a middle layer of mRNA, and an outer layer of PPH polymer. This LbL nanoparticle demonstrates a high efficiency in mRNA transfection across various hard-to-transfect cancer cells. It holds promise as a nanocarrier candidate for cancer mRNA therapy, while also offering MRI capabilities. Significantly, the LbL nanoparticle with a weakly-charged SPION core outperforms those with negatively charged and highly cationic cores in terms of mRNA release and transfection efficiency, suggesting the crucial role of the weak cationic charge in the nanoparticle core for successful mRNA delivery in this specific context.

## 2. Materials and Methods

### 2.1. Materials

All chemicals were purchased from Sigma-Aldrich (St. Louis, MO, USA) unless otherwise stated. Chitosan (2.3 kMW) was obtained from Acmey Industrial (Shanghai, China). Branched PEI (MW 2 kDa) was purchased from Polysciences (Warrington, PA, USA). CleanCap EGFP mRNA was purchased from TriLink Biotechnologies (San Diego, CA, USA). Low molecular weight heparin used in PPH polymer was purchased from Galen Laboratory Supplies (North Haven, CT, USA). Furthermore, (1-Ethyl-3-[3-dimethylaminopropyl] carbodiimide hydrochloride) (EDC), N-hydroxysuccinimide (NHS), Lab-Tek II 8-well chambered coverglass, NucBlue DAPI reagent, Lipofectamine 2000, LysoTracker Red DND-99, ultrapure agarose, antibiotic-antimycotic (100×), Tryple Express Enzyme solution, RPMI 1640, and DMEM cell culture medium were purchased from Invitrogen (Carlsbad, CA, USA). HyClone characterized fetal bovine serum (FBS) was purchased from GE Healthcare Life Sciences (Pittsburgh, PA, USA). Label IT Tracker Intracellular Nucleic Acid Labeling Kits were purchased from Mirus Bio (Madison, WI, USA). Cyanine5 NHS ester was purchased from Lumiprobe Corporation (Cockeysville, MD, USA). SpectraPOR7 dialysis membrane was purchased from Repligen Corp (Waltham, MA, USA). Moreover, 4T1, HepG2 and C6 cell lines were purchased from American Type Culture Collection (Manassas, VA, USA). 

### 2.2. Nanoparticle Synthesis

Iron oxide nanoparticles were produced by thermal decomposition and coated with crosslinked silane-PEG (IOSPM), or by coprecipitation and coated with various polymer formulations including catechol-modified, chitosan-grafted PEG (IOCCP) and 2 kDa/25 kDa MW PEI conjugated CCP (IOCCP-2k PEI/IOCCP-25k PEI) as previously reported [19,20]. Briefly, for the thermal decomposition for IOSPM synthesis, hydrophobic oleic acid-coated iron oxide nanoparticles were initially synthesized from an Fe-oleate precursor. A ligand exchange process was employed to remove the oleic acid coating and graft a silanized PEG monolayer to the iron oxide surface. The resulting product was water-soluble iron oxide nanoparticles coated with a silanized PEG monolayer (IOSPM). IOCCP/IOCCP-2k PEI/IOCCP-25k PEI were synthesized by coprecipitation of iron chlorides (2:1 ratio of Fe^3+^ to Fe^2+^) and polymers (CCP/CCP-2k PEI/CCP-25k PEI) through the addition of an ammonia solution in an aqueous environment. For CCP synthesis, 2300 MW of purified chitosan and aldehyde-activated methoxy PEG were reacted via reductive amination, followed by the functionalization with the catechol group by reacting 3,4-dihydroxybenzaldehyde and the copolymer via reductive amination. Furthermore, 2 kDa/25 kDa MW PEI functionalized with Traut’s reagent and CCP functionalized with succinimidyl iodoacetate were reacted to form the CCP-2k/25k PEI copolymer via thioester linkage.

### 2.3. Synthesis of Perfluorinated PEI (PP)

Perfluoroheptanoic acid (PFHA), as a fluorocarbon moiety, was conjugated onto PEI via EDC/NHS coupling chemistry. PFHA, EDC, and NHS were separately dissolved in methanol at 50 mg/mL concentration. PFHA, EDC, and NHS solutions at the molar ratio of 1:1.2:1.44 were mixed by adding EDC and subsequently NHS to the PFHA solution. The mixed solution was incubated for 3 h on a rocker at room temperature. Subsequently, branched 2k PEI was dissolved in methanol at 50 mg/mL and added to the PFHA-EDC-NHS mixture solution at the molar ratio of 1:7 (PEI:PFHA) and reacted at room temperature for 16 h. The resultant solution was dialyzed against Milli-Q water for 2 days using 1k MWCO SpectraPOR7 dialysis membrane. The dialyzed solution (PP) was freeze-dried before redissolution.

### 2.4. Formation of NP-mRNA-PPH Complex

NPs (IOSPM/IOCCP/IOCCP-2k PEI/IOCCP-25k PEI) were all dissolved in 20 mM HEPES buffer (pH 7.4) at the concentration of 0.1 mg Fe/mL. mRNA was diluted to 0.5 mg/mL in 20 mM HEPES buffer (pH 7.4). PP powder was redissolved in 20 mM HEPES buffer (pH 7.4) at 3.75 mg/mL. Low molecular weight heparin was dissolved in 20 mM HEPES buffer (pH 7.4) at 0.25 mg/mL concentration. To make an NP-mRNA-PPH complex, 5 μL of NP was loaded into a Hamilton microliter syringe and injected (at the flow rate of 0.5 μL/s controlled by a syringe pump) into a 5 μL mRNA solution, and stirred by a rotor tip at 500 RPM for homogenous mixing. Next, the resulting 10 μL NP-mRNA was loaded into a Hamilton microliter syringe and slowly injected into 10 µL of spinning PP solution at the flow rate of 1 μL/s. Furthermore, 10 μL heparin was subsequently loaded into a Hamilton microliter syringe and slowly injected at the flow rate of 0.5 μL/s into the 20 μL NP-mRNA-PP solution to form NP-mRNA-PPH complexes.

### 2.5. Hydrodynamic Size, Serum Stability, and Zeta Potential Measurement

The hydrodynamic size and zeta potential of the NP cores and NP-mRNA-PPH complexes were determined by dynamic light scattering (DLS) using a Zetasizer Nano-ZS (Malvern Instruments, Worcestershire, UK). For the size stability study, (IOCCP)-mRNA-PPH samples were freshly prepared within an hour or stored at 4 °C fridge for 7 days for size measurement by DLS. The measurements were performed in 20 mM HEPES buffer (pH 7.4) at room temperature. To test serum stability of the samples, the samples were diluted 100 times with DMEM or RPMI 1640 cell culture medium (supplemented with 10% fetal bovine serum (FBS) and 1% antibiotic-antimycotic) and placed in a 37 °C water bath. Hydrodynamic size measurements were conducted every day within 2 weeks.

### 2.6. mRNA Encapsulation Efficiency Study

Free mRNA, (IOSPM)-mRNA-PPH, (IOCCP)-mRNA-PPH, (IOCCP-2k PEI)-mRNA-PPH, and (IOCCP-25k PEI)-PPH samples containing 0.5 μg of mRNA were incubated with 0.2 μg ethidium bromide for 5 min at room temperature before being subjected to fluorescence analysis using a SpectraMax i3 microplate reader (Molecular Devices, Sunnyvale, CA, USA) with 260 nm excitation and 590 nm emission. To account for background noise, the fluorescence intensity of each sample was adjusted by subtracting the determined background signal of 0.2 μg ethidium bromide. The encapsulation efficiency was determined by dividing the fluorescence intensity of a sample by that of free mRNA. 

### 2.7. Gel Electrophoresis Retardation Assay

Free mRNA, (IOSPM)-mRNA-PPH, (IOCCP)-mRNA-PPH, (IOCCP-2k PEI)-mRNA-PPH, and (IOCCP-25k PEI)-PPH samples (with and without 12.5 μg heparin added) were added to 1% agarose gel at 0.5 μg mRNA per lane, and 120 V of electric potential was applied across the gel for 30 min at 120 V. Gels were stained with 0.5 μg/mL ethidium bromide and visualized using a Bio-Rad Universal Hood II Gel Doc System.

### 2.8. Transmission Electron Microscopy (TEM) Imaging

5 μL of (IOSPM)-mRNA-PPH, (IOCCP)-mRNA-PPH, (IOCCP-2k PEI)-mRNA-PPH, or (IOCCP-25k PEI)-PPH solution was added to Formvar/carbon-coated 300-mesh copper grid (Ted Pella, Inc., Redding, CA, USA) and left to dry overnight. The TEM samples were imaged using a Tecnai G2 F20 electron microscope (FEI, Hillsboro, OR, USA) at a voltage of 200 kV.

### 2.9. Fourier-Transform Infrared (FTIR) Spectra Analysis

Potassium bromide was mixed with the lyophilized samples (1 wt.%) and then pressed into pellets. Pellet samples were analyzed using a Nicolet 6700 FTIR spectrometer to obtain FTIR spectra, with a resolution of 4 cm^−1^ and an average of 64 runs. The scanning range was from 400 to 3900 cm^−1^_._

### 2.10. Cell Culture

4T1 mouse breast cancer cells were cultured in RPMI1640 medium supplemented with 10% *v*/*v* FBS and 1% *v*/*v* antibiotic–antimycotic. HepG2 human liver cancer cells and C6 rat glioma cells were cultured in DMEM medium supplemented with 10% *v*/*v* FBS and 1% *v*/*v* antibiotic–antimycotic. Cultures were maintained in a 37 °C and 5% CO_2_ humidified incubator.

### 2.11. Cellular Uptake and Endosomal Escape Studies of mRNA-PPH

For the mRNA-PPH cellular uptake study, PP was labeled with Cy5-NHS fluorophore by mixing 100 µL of PP (10 mg/mL) with 58.7 µL Cy5-NHS fluorophore (250 mM in DMSO). The mixture reacted for 30 min under gentle rocking at room temperature in the dark. The unreacted Cy5-NHS molecules were removed via dialysis in the Milli-Q water using 1k MWCO SpectraPOR7 dialysis membrane. To form the labeled mRNA-PPH complex, 5 μL mRNA (0.5 μg/μL) was loaded into a Hamilton microliter syringe and slowly injected at the flow rate of 1 μL/s into 5 μL of PP solution (7.5 μg/μL), stirred by a rotor tip at 500 RPM. Furthermore, 10 μL heparin was subsequently loaded into a Hamilton microliter syringe and slowly injected at the flow rate of 0.5 μL/s into the 10 μL mRNA-PP solution. Furthermore, 4T1, HepG2, and C6 cells were seeded at 8000, 30,000, and 8000 cells per well, respectively, in 8-well glass chambers. After 24 h, labeled mRNA-PPH was then added to cells at 2 μg/mL mRNA concentration and incubated for 8 h. All cells were then washed three times with cold PBS and fixed with paraformaldehyde (4% in PBS) for 15 min at room temperature. The fixed cells were further stained by WGA-555 and NucBlue FixCell ReadyProbe DAPI reagent with cold PBS washing three times before each staining. Confocal images were acquired using a Leica SP8X confocal laser scanning microscope (Wetzlar, Germany).

For mRNA-PPH endosomal escape study, mRNA was labeled with Cy5 following the manufacturer’s protocol for the Label IT Tracker Intracellular Nucleic Acid Labeling Kit. Labeled mRNA was complexed into mRNA-PPH in the same manner as mRNA-PPH synthesized in the mRNA-PPH cellular uptake study. Furthermore, 4T1 and HepG2 were seeded at 10,000 and 30,000 cells per well, respectively, in 8-well glass chambers. After 24 h, labeled mRNA-PPH was then added to cells at 2 μg/mL mRNA concentration, incubated for 10 h before adding 75 nM of Lysotracker Red DND reagent, and then incubated for another 1 h. All cells were then washed three times with cold PBS and fixed with paraformaldehyde (4% in PBS) for 15 min at room temperature. The fixed cells were further washed with cold PBS three times. NucBlue FixCell ReadyProbe DAPI reagent was diluted 10 times in cold PBS and 100 μL was added to each well. Confocal images were acquired using a Leica SP8X confocal laser scanning microscope (Wetzlar, Germany).

### 2.12. Cellular Uptake and Endosomal Escape Studies of NP-mRNA-PPH

mRNA was labeled with Cy5 following the manufacturer’s protocol for the Label IT Tracker Intracellular Nucleic Acid Labeling Kit. Labeled mRNA was complexed into four NP-(mRNA)-PPHs in the same manner as unlabeled NP-mRNA-PPH. Furthermore, 4T1, HepG2, and C6 cells were seeded at 10,000, 30,000, and 10,000 cells per well, respectively, in 8-well glass chambers. After 24 h, four labeled NP-mRNA-PPHs were then added to cells at 2 μg/mL mRNA concentration, incubated for 10 h before adding 75 nM of Lysotracker Red DND reagent, and then incubated for another 1 h. All cells were then washed three times with cold PBS and fixed with paraformaldehyde (4% in PBS) for 15 min at room temperature. The fixed cells were further washed with cold PBS three times. NucBlue FixCell ReadyProbe DAPI reagent was diluted 10 times in cold PBS and 100 μL was added to each well. Confocal images were acquired using a Leica SP8X confocal laser scanning microscope (Wetzlar, Germany).

### 2.13. mRNA Release Study

mRNA was labeled with Cy3 following the manufacturer’s protocol for the Label IT Tracker Intracellular Nucleic Acid Labeling Kit. NP (IOSPM, IOCCP, IOCCP-2k PEI, or IOCCP-25k PEI) was tagged with Cy5 by reacting 2 μL (5 mg/mL) NHS-Cy5 with 1 mg/mL NP solution (in 20 mM HEPES buffer, pH 7.4) before 30 min incubation and purification via size exclusion chromatography PD-10 column packed with S-200 resin. Labeled mRNA and NPs were complexed into four NP-(mRNA)-PPHs in the same manner as unlabeled NP-mRNA-PPH. Furthermore, 4T1, HepG2, and C6 cells were seeded at 10,000, 30,000, and 10,000 cells per well, respectively, in 8-well glass chambers. After 24 h, four labeled NP-mRNA-PPHs were then added to cells at 2 μg/mL mRNA concentration, and incubated for 10 h. All cells were then washed three times with cold PBS and fixed with paraformaldehyde (4% in PBS) for 15 min at room temperature. The fixed cells were further washed with cold PBS three times. NucBlue FixCell ReadyProbe DAPI reagent was diluted 10 times in cold PBS and 100 μL was added to each well. Confocal images were acquired using a Leica SP8X confocal laser scanning microscope (Wetzlar, Germany). 

### 2.14. In Vitro Cell Transfection

4T1 and C6 cells were seeded at 1500 cells per well in 96-well plates. HepG2 was seeded at 8000 cells per well in 96-well plates. After 24 h, (IOSPM)-mRNA-PPH, (IOCCP)-mRNA-PPH, (IOCCP-2k PEI)-mRNA-PPH, and (IOCCP-25k PEI)-mRNA-PPH were added to 100 μL of fully supplemented culture medium to give a final mRNA concentration of 2 μg/mL in each well for all cancer cell lines. Lipofectamine 2000-mRNA complexes were added to each well according to manufacturer’s protocol as a control. The cells were incubated with complexes for 48 h and were imaged with a Nikon TE300 inverted fluorescent microscope (Tokyo, Japan).

### 2.15. Cell Viability Studies

4T1, HepG2, and C6 cells were seeded at 4000, 15,000, and 4000 cells per well in 96-well plates, respectively. After 24 h, the cells were treated with (IOSPM)-mRNA-PPH, (IOCCP)-mRNA-PPH, (IOCCP-2k PEI)-mRNA-PPH, (IOCCP-25k PEI)-mRNA-PPH, or Lipofectamine 2000-mRNA at mRNA concentration of 1 μg/mL. The cells were treated for 24 h before the cell viability was determined using the Alamar Blue assay. The fluorescent signal readout was obtained by using a SpectraMax i3 microplate reader (Molecular Devices, Sunnyvale, CA, USA) with 550 nm excitation and 590 nm emission. The fluorescence intensities of all the treatment groups were normalized so that the viability of the untreated cell group was 100%.

### 2.16. Statistical Analysis

The results are presented as mean values ± standard error of the mean. The statistical differences were determined by one-way ANOVA post hoc tests. The values were considered statistically significant at *p* < 0.05.

## 3. Results and Discussion

### 3.1. NP Core and NP-mRNA-PPH Complex Design

**Figure 1:**
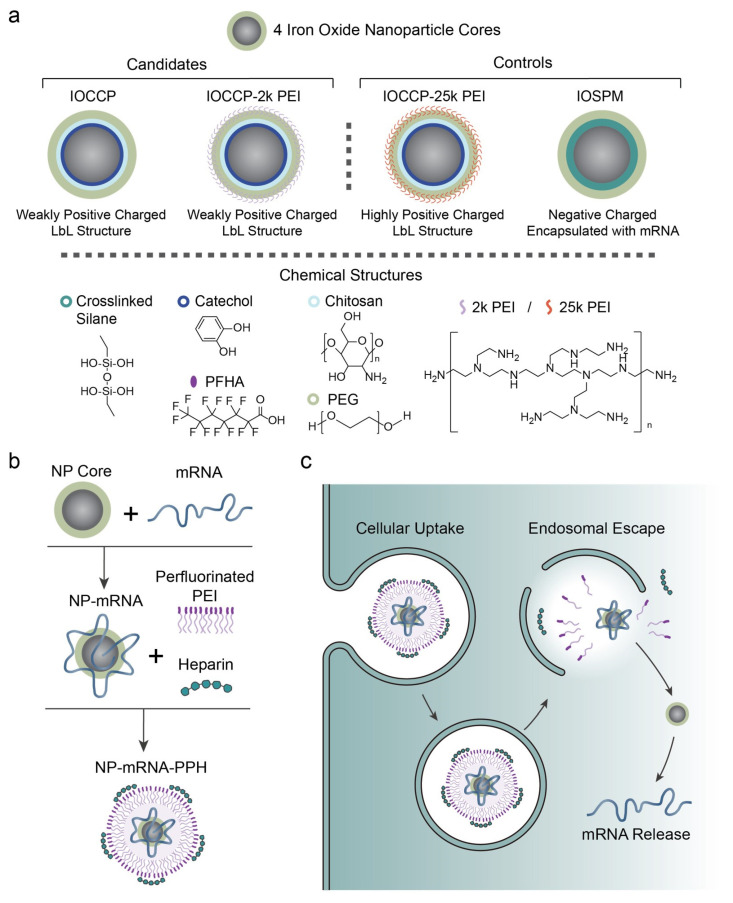
Nanoparticle core/PPH shell mRNA nanocarriers for mRNA delivery. (**a**) Schematic illustration of the chemical structures of four nanocarriers investigated in this study. (**b**) Synthesis process to form NP-mRNA-PPH. (**c**) Schematic illustration of the key steps in delivering mRNA to cancer cells by the nanocarrier.

In this study, we investigated SPIONs with four different surface coatings (IOCCP, IOCCP-2k PEI, IOCCP-25k PEI, and IOSPM) as cores (NPs) to complex mRNA (Figure 1a). Among them, IOCCP and IOCCP-2k PEI were chosen as NP candidates due to their weakly cationic natures, which may facilitate mRNA release due to their weak association with mRNA. As a control for comparison, IOCCP-25k PEI, a highly cationic coating, was selected with the hypothesis that it would lead to poor transfection due to difficulties in releasing mRNA. Additionally, the necessity of using the LbL structure was examined by investigating anionic IOSPM, which could be encapsulated with mRNA in the complex. The superparamagnetic properties and synthesis methods of these NPs were described previously [19,20]. In terms of composition, IOCCP is an iron oxide core coated with chitosan-PEG copolymer modified with catechol, while IOCCP-2k/25k PEI is similar but coated with an extra layer of 2 kDa/25 kDa PEI. IOSPM consists of an iron oxide core coated with silanized PEG, resulting in negative charges due to the presence of silanol (-Si-O-) groups. The composition of the polymer components on each NP was confirmed through FTIR analysis (Figure S2).

To form a nanoscale complex, NP, mRNA, and PPH were assembled through sequential deposition using the following steps: (1) mRNA onto NP core to form NP-mRNA, and (2) PPH onto NP-mRNA to form NP-mRNA-PPH (Figure 1b). A successful NP-mRNA-PPH complex is anticipated to undergo cellular uptake, escape from the endosome prior to its maturation into late endosomes and lysosomes, and ultimately release the mRNA for protein translation (Figure 1c).

### 3.2. Physicochemical Property Characterization

Physicochemical properties, including size and surface charge, play a critical role in nanoparticle-mediated delivery application as they influence nanoparticles’ stability in the bloodstream and their interaction with cells. For in vivo applications, the size of nanoparticles is a critical consideration. Nanoparticles larger than 200 nm tend to activate the complement system, which results in their rapid clearance from the bloodstream and accumulation in the liver and spleen. On the other hand, nanoparticles that are too small (less than 10 nm) can be swiftly eliminated by the kidneys [21]. Thus, achieving an optimal size range is essential to ensure prolonged circulation and effective delivery of nanoparticles in vivo. Furthermore, it has been reported that tumor cells exhibit higher uptake rates of cationic nanoparticles compared to anionic ones [22]. This observation suggests that the positive charge on cationic nanoparticles may enhance their cellular internalization in tumor cells, potentially leading to improved delivery and therapeutic efficacy in cancer treatment.

**Figure 2:**
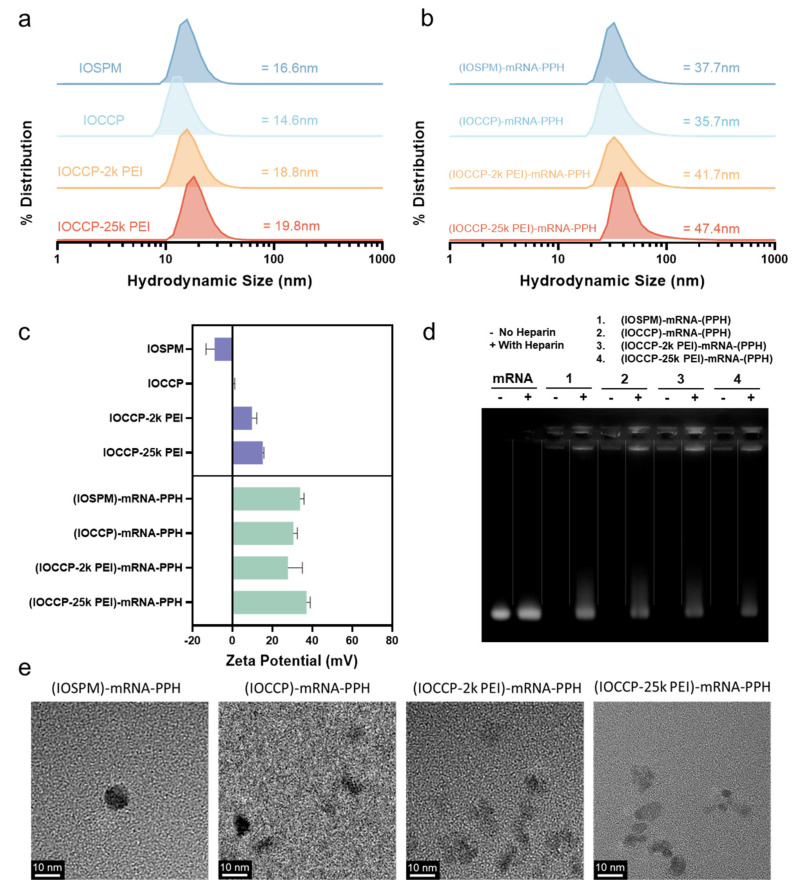
Physicochemical characterization of NP cores and NP-mRNA-PPH complexes. (**a**) Number distributions of hydrodynamic sizes of NP cores, including IOSPM, IOCCP, IOCCP-2k PEI, and IOCCP-25k PEI. (**b**) Number distributions of hydrodynamic sizes of NP-mRNA-PPH complexes, including (IOSPM)-mRNA-PPH, (IOCCP)-mRNA-PPH, (IOCCP-2k PEI)-mRNA-PPH, and (IOCCP-25k PEI)-mRNA-PPH. (**c**) Zeta potential measurements of NP cores and NP-mRNA-PPH complexes. (**d**) Gel retardation assay of the four NP-mRNA-PPH complexes. For lanes with a negative sign (i.e., ‘−’) on top, no heparin was added. For lanes with a positive sign (i.e., ‘+’) on top, excessive heparin was added to materials for complex disassembly or mRNA control. (**e**) TEM images of NP-mRNA-PPH complexes.

The hydrodynamic sizes and zeta potentials of four NP cores and their corresponding NP-mRNA-(PPH) complexes were assessed using dynamic light scattering (DLS). The four NP cores (IOSPM, IOCCP, IOCCP-2k PEI, IOCCP-25k PEI) exhibited similar sizes in the range of 14–20 nm (Figure 2a). Hydrodynamic sizes of four mRNA-loaded complexes were also comparable. The (IOSPM)-mRNA-PPH and (IOCCP)-mRNA-PPH had sizes of 37.7 nm and 35.7 nm, respectively. On the other hand, the complexes containing PEI, i.e., (IOCCP-2k PEI)-mRNA-PPH and (IOCCP-25k PEI)-mRNA-PPH, were slightly larger in size, measuring 41.7 nm and 47.4 nm, respectively. TEM images revealed that NP-mRNA-PPH complexes exhibited roughly spherical morphology, with sizes of around 10 nm in dry conditions. The small sizes of these NP-mRNA-PPHs indicate their potential for prolonged circulation in vivo. NP-mRNA-PPHs were shown to remain structurally stable in two serum-containing cell media over a period of 14 days (Figure S3).

The zeta potentials of the four NP cores were intentionally designed to vary significantly. The anionic core, IOSPM, had a zeta potential of −8.9 mV, while the zeta potential of IOCCP was close to neutral at 0.29 mV. The presence of amine groups in PEI resulted in positively charged coating polymers on the iron oxide NPs, yielding zeta potential values of 9.78 mV for IOCCP-2k PEI and 15.17 mV for IOCCP-25kPEI. As the NP cores were subsequently encapsulated with negatively charged mRNA to form complexes, the difference in core charge was expected to influence the release profile of mRNA during transfection. It should be noted that the surface charge of the NP-mRNA-PPH complexes is primarily determined by the outer layer of positively charged PPH. All four NP-mRNA-PPH complexes exhibited a positive charge of ~30 mV. The positive zeta potentials of these complexes lay the foundation for preferential cellular uptake and endosomal escape, facilitating effective transfection in cancer cells. 

A gel retardation assay was performed to confirm the successful encapsulation and protection of mRNA within the NP-mRNA-PPHs complexes (Figure 2d). No mRNA band was observed in any of four NP-mRNA-PPH complexes, indicating that the mRNA was effectively encapsulated by the complexes. In contrast, the band corresponding to naked mRNA was clearly visible. To further investigate the integrity of encapsulated mRNA, excessive heparin was added to the NP-mRNA-PPH samples. Heparin is known to disrupt the electrostatic interactions between cationic and anionic molecules, potentially leading to the disassembly of complexes. In the heparin-loaded samples, the mRNA was released from the NP-mRNA-PPH complexes and migrated the same distance as naked mRNA, indicating that the encapsulated mRNA remained intact in the nanocomplexes. As a control, the same amount of heparin was added to naked mRNA, demonstrating that heparin had negligible influence on the integrity of mRNA alone. Furthermore, the mRNA encapsulation efficiency and mRNA loading on each NP-mRNA-PPH complex were measured and calculated. It was found that approximately 80% of mRNA was fully encapsulated within all NP-mRNA-PPH complexes. The encapsulated mRNA accounted for 4–5% of the total mass of the complexes (Table S1). 

### 3.3. Cellular Uptake and Endosomal Escape Studies

The cellular uptake and endosomal escape of the four NP-mRNA-PPH complexes were investigated, as these steps are crucial for successful transfection. Cellular uptake involves the adherence of the nanoparticle to the cell membrane and subsequent internalization through endocytosis [23]. Once inside the cell, the nanoparticle must escape from the endosome before it matures into a late endosome, where degradation can occur. 

**Figure 3:**
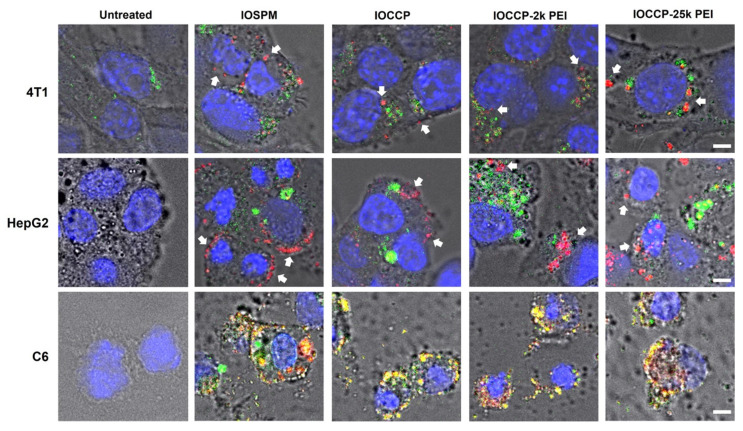
Confocal fluorescence images of three different cell lines subjected to various treatments by NP-mRNA-PPH complexes of different formulations illustrating the cellular uptake and endosomal escape studies conducted in this investigation. The NP-mRNA-PPH complexes are named according to their core materials (top row). mRNA was labeled with Cy5 fluorophores before conjugated to NP-mRNA-PPH complexes. NP-mRNA-PPH complexes were added to cell cultures at 2 μg/mL mRNA dosage, and the cells were incubated for 10 h at 37 °C and then incubated for an additional one hour after adding Lysotracker Red reagent. mRNA-Cy5 signals are presented in red, Lysotracker in green, DAPI nuclear stain in blue, with bright field images shown as the background. White arrows indicate the signals of the NP-mRNA-PPH complexes separated from the endo-lysosomes. Scale bar is 5 μm.

Three representative hard-to-transfect cancer cell lines were selected for this study, including triple negative breast cancer cell 4T1, liver cancer cell HepG2, and glioma cell C6 [24,25,26]. Fluorescence microscopy was used to observe the spatial distribution of the complexes upon delivery to cancer cells. For this purpose, mRNA was labeled with Cy5 fluorophores (in red) before complexation in the NP-mRNA-PPHs. The cell nuclei were stained with DAPI (in blue). The images captured using fluorescence microscopy (Figure 3) revealed that all four NP-mRNA-PPH complexes were able to penetrate the plasma membrane and reach the cytoplasm of 4T1, HepG2, and C6 cells. 

The accumulation of mRNA in the (IOSPM)-mRNA-PPH complex near the cell membrane of HepG2 and C6 cells deserves further discussion. As both the negatively charged IOSPM and mRNA were encapsulated inside the positively charged outer layer of the PPH polymer, there could have been competition between IOSPM and mRNA for electrostatic association with PPH. This competition might weaken the binding between mRNA and PPH, potentially compromising the LbL structure of NP-mRNA-PPH complex and its ability to efficiently transport mRNA into the cells. This finding highlights the importance of maintaining a stable LbL structure for the association between mRNA and the protective layer. 

Furthermore, the lower amount of NP-mRNA-PPH complexes observed within 4T1 cells compared to the other two cell lines (HepG2 and C6) could potentially result in lower transfection efficiency in 4T1 cells. This difference in cellular uptake could be attributed to the fact that three cancer cell lines express different sets of surface receptors; it is likely that the surface receptors present on each cell type play a role in mediating the cellular uptake of NP-mRNA-PPH complexes in a cell-type specific manner. In addition, it is known that various cells employ different mechanisms of endocytosis, such as clathrin-mediated endocytosis, caveolin-mediated endocytosis, clathrin/caveolae-independent endocytosis, and micropinocytosis [27]. The disparity in endocytosis mechanisms among cell types could potentially impact the rate of cellular uptake of NP-mRNA-PPH complexes.

To evaluate the abilities of NP-mRNA-PPH complexes to escape from endo-lysosomes, lysotracker (in green) was added to trace the location of endo-lysosomes. After 10 h from transfection, a clear separation between the NP-mRNA-PPH complexes and endo-lysosomes was observed in 4T1 and HepG2 cells for all four complexes, indicating successful endosomal escape. In these two cell lines, there was no noticeable difference in the endosomal escape efficiency among the four NP-mRNA-PPH complexes. This successful endosomal escape can be attributed to the presence of PEI in the outer shell layer of the complexes, as PEI is known to facilitate endosomal escape through the proton sponge effect [28]. However, in C6 cells, the signals from the lysotracker and mRNA could not be distinguished clearly (appearing as yellow in the confocal images shown in Figure 3). This could be attributed to the relatively small cytoplasmic space observed in C6 cells as compared to the other two cell lines and their higher activity of endocytosis, as indicated by a denser endosome signal per cell. These factors might have made it challenging to distinguish the separation between NP-mRNA-PPH complexes and endo-lysosomes in C6 cells. 

### 3.4. mRNA Release Study

The release of mRNA is a critical step for successful mRNA transfection. We have demonstrated earlier that mRNA-PPH complexes exhibit favorable mRNA release profiles (Figure S1). However, in the case of NP-mRNA-PPH complexes, the presence of NP cores with different surface charges can affect the electrostatic interactions with mRNA, potentially leading to different mRNA release behaviors among the four nanoparticle formulations.

**Figure 4:**
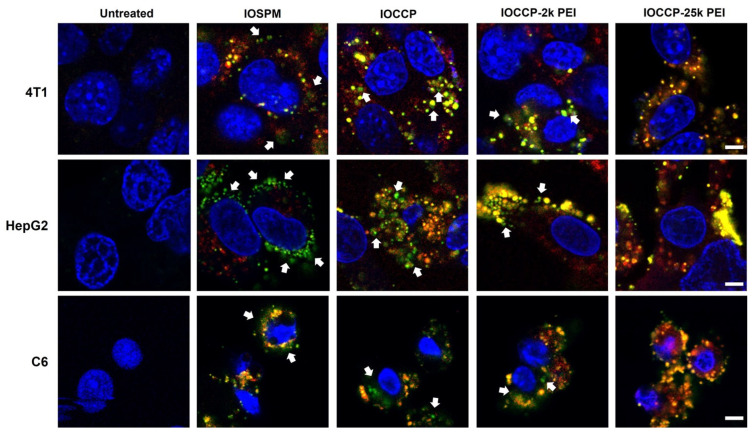
mRNA release study of three cancer cell lines at the 10 h time point. NP-mRNA-PPH complexes, composed of different NP cores (IOSPM, IOCCP, IOCCP-2k PEI, or IOCCP-25k PE) and mRNA labeled with Cy5 and Cy3, respectively, were incubated with the three cancer cell lines (4T1, HepG2, and C6). The fluorescence images depict the spatial distribution of released mRNA (green) and NP cores (red) within the cells. Blue indicates DAPI nuclear staining. White arrows indicate the representative locations where mRNA is released from or bound with NP cores. Scale bar is 5 μm.

To track the mRNA release from NP-mRNA-PPH, the mRNA and NP cores were labeled with Cy3 (green) and Cy5 (red), respectively, before complexation to form NP-mRNA-PPH complexes. The four dual-labeled NP-mRNA-PPH complexes were added separately to each of the three cancer cell lines (4T1, HepG2, and C6) and incubated for 10 h. Fluorescent images were captured to visualize the mRNA release process (Figure 4). Separation of signals indicates successful mRNA release, while overlapping signals (yellow) indicate bound mRNA within the NP-mRNA-PPH complexes. The surface charge of the NP core influenced the mRNA release, with (IOSPM)-mRNA-PPH demonstrating excellent mRNA release, particularly in HepG2 cells. 

The different behaviors of mRNA release from NP-mRNA-PPH complexes can be attributed to the electrostatic interactions between the NP cores and mRNA. In the case of (IOSPM)-mRNA-PPH, the negative charge of IOSPM repels mRNA, leading to efficient mRNA release. However, the compromised binding between mRNA and PPH observed in the cellular uptake study prevents effective internalization of the released mRNA, resulting in its accumulation near the cellular membrane. In contrast, (IOCCP)-mRNA-PPH exhibited a well-tuned electrostatic interaction between the neutrally charged IOCCP and mRNA. This led to a prominent signal overlap between mRNA and NP in the cytoplasm in both IOCCP-2k PEI- and IOCCP-25k PEI-treated groups across all three cancer cells, indicating that mRNA remained mostly bound to NP cores. 

Both IOCCP-2k PEI and IOCCP-25k PEI, which are cationic in nature, strongly interacted with mRNA, resulting in a substantial energy barrier for mRNA release. The highly charged IOCCP-25k PEI exhibited a stronger interaction, leading to greater retention of mRNA within the NP complexes. However, IOCCP-2k PEI, with a lower overall charge compared to IOCCP 25k PEI, showed a more sufficient release of mRNA in 4T1 and C6 cells.

These observations collectively demonstrate that the properties of the core NP in NP-mRNA-PPH complexes play a critical role in mRNA release. Negatively charged IOSPM and neutrally charged IOCCP demonstrated superior mRNA release compared to positively charged IOCCP-2k PEI and IOCCP-25k PEI. Furthermore, cell line-dependent mRNA release was observed in 4T1 cells, indicating that different cell types may respond differently to the NP-mRNA-PPH complexes. This suggests that the choice of NP core and formulation should be optimized based on the specific characteristics and requirements of the target cell type.

### 3.5. In Vitro mRNA Transfection

Following the mRNA release study shown above, we conducted in vitro mRNA transfection using the four NP-mRNA-PPH complexes on 4T1, HepG2, and C6 cancer cells. To assess the transfection efficiency of NP-mRNA-PPH, we utilized capped enhanced green fluorescent protein (EGFP) mRNA as the reporter gene mRNA. In this experiment, cells transfected using the commercially available transfection reagent Lipofectamine 2000 served as a positive control, while untreated cells served as the negative control. 

**Figure 5:**
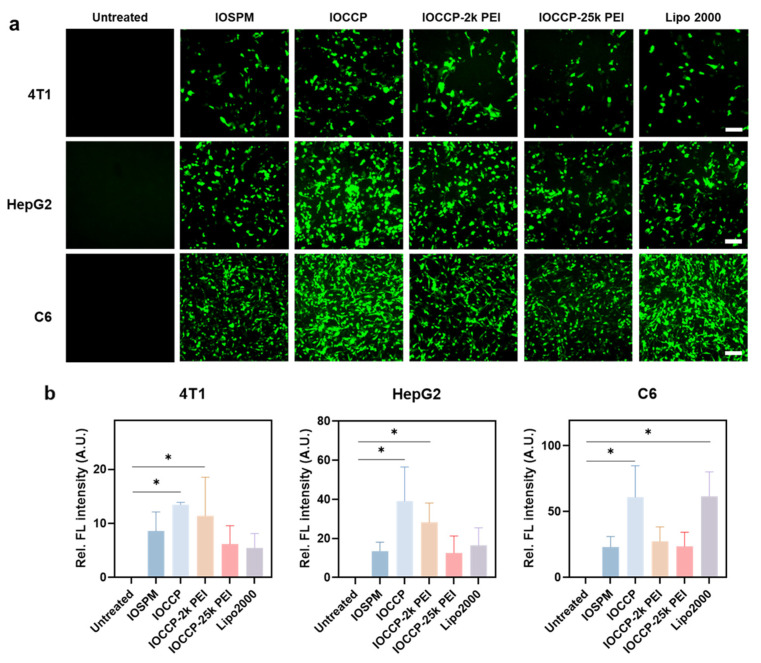
mRNA transfection efficiency in three cancer cell lines. NP-mRNA-PPH complexes are denoted by their core materials, which include (IOSPM)-mRNA-PPH, (IOCCP)-mRNA-PPH, (IOCCP-2k PEI)-mRNA-PPH, and (IOCCP-25k PEI)-mRNA-PPH. (**a**) Images of three cell lines (4T1, HepG2, and C6) transfected with various NP-mRNA-PPH complexes as well as lipofectamine 2000-mRNA as a positive control. Scale bar is 100 μm. (**b**) Quantification of relative fluorescence intensity of the cell transfection images. * *p* < 0.05.

Fluorescence micrographs of cells were captured after transfection and the transfection results were quantitatively analyzed using the relative fluorescence intensities (Figure 5 and Figure S4). (IOCCP)-mRNA-PPH achieved the highest transfection efficiency among three cancer cells, compared to other NP-mRNA-PPHs. In 4T1 and HepG2 cells, (IOCCP)-mRNA-PPH exhibited higher transfection efficiency than Lipofectamine 2000, while in C6 cells, the transfection efficiency of (IOCCP)-mRNA-PPH was comparable to that of Lipofectamine 2000. The superior transfection efficiency of (IOCCP)-mRNA-PPH can be attributed to its favorable cellular uptake, endosomal escape, and mRNA release properties. On the other hand, the transfection efficiencies of (IOCCP-2k PEI)-mRNA-PPH and (IOCCP-25k PEI)-mRNA-PPH were lower than that of (IOCCP)-mRNA-PPH across all three cell lines. Consistent with the earlier findings on mRNA release (Figure 4) where (IOCCP)-mRNA-PPH exhibited greater mRNA release than (IOCCP-2k PEI)-mRNA-PPH, which in turn released mRNA more readily than (IOCCP-25k PEI)-mRNA-PPH, the transfection efficiency followed a similar trend. As the molecular weight of the PEI coated on IOCCP increased, the transfection efficiency decreased. Although (IOSPM)-mRNA-PPH showed considerable mRNA release, its transfection performance was lower than that of (IOCCP)-mRNA-PPH. This observation agrees with a reported finding, where reduced mRNA transfection efficiency was associated with an increased amount of SPIONs co-delivered with mRNA using a lipid nanocarrier [29]. However, the mechanism underlying this phenomenon was not discussed in their study. In the case of (IOSPM)-mRNA-PPH, the lower transfection efficiency may be attributed to the majority of mRNA being located near the plasma membrane, resulting in lower mRNA availability for translation in the cytoplasm. The absence of signal in untreated cells confirmed that the EGFP transfection results were not affected by autofluorescence from the cancer cells.

Noticeably, EGFP expression in C6 cells was the highest among the three cell lines. The observed high EGFP expression in C6 cells can be attributed to their rapid proliferation rate, which leads to an increased demand for protein synthesis [30]. Consequently, C6 cells have high translational activity, making them more receptive to exogenous mRNA delivered through transfection. 

On the other hand, the transfection efficiency in 4T1 cells was relatively lower than those on HepG2 and C6 cell transfection for the four NP-mRNA-PPHs complexes. This finding aligns with the earlier observations that cellular uptake and the mRNA release activities were less active in 4T1 cells. In our previous study on using a different NP for DNA delivery in breast cancer cells, DNA release was suppressed in 4T1 cells when compared to SKBR3 and MCF7 breast cancer cells [31]. This suggests that there may be intracellular factors in 4T1 cells that hinder the release of nucleic acids from nanocarriers. Further investigation into these factors is necessary to improve the transfection efficiency in 4T1 cells.

The storage stability of the freshly prepared optimal transfection agent (IOCCP)-mRNA-PPH in HEPES solution and under refrigeration at 4 °C for 7 days was also investigated by size measurement (Figure S5). It was found that the size of (IOCCP)-mRNA-PPH stored at 4 °C over 7 days showed a comparable size distribution to the freshly prepared samples. This suggests that it may not be necessary to generate the nanocomposite for every use if it can be stored under refrigeration for a certain period of time.

### 3.6. Biocompatibility Evaluation

In addition to transfection efficiency, biocompatibility is another critical factor for evaluating mRNA nanocarriers. Cationic moieties in mRNA nanocarriers can have both positive and negative effects. On one hand, they can enhance transfection efficiency by facilitating efficient mRNA condensation and protection. On the other hand, they can potentially inflict undesirable cytotoxicity by disrupting cellular membranes and generating reactive oxygen species [32,33]. Hence, achieving an optimal balance between biocompatibility and transfection efficiency is essential in the design of NP-mRNA-PPH complexes.

**Figure 6:**
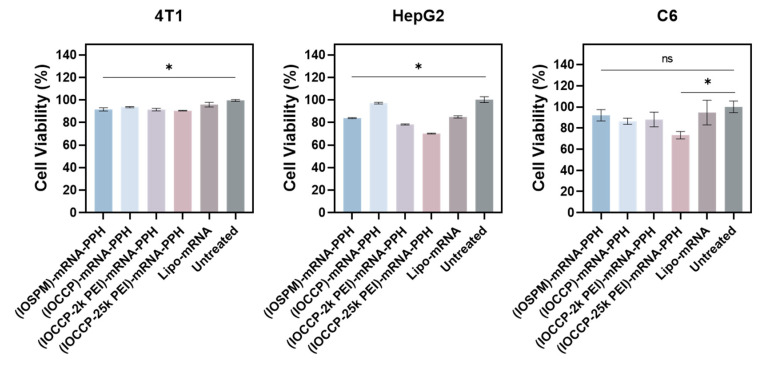
Cell viability assessment of three cancer cell types treated by (IOSPM)-mRNA-PPH, (IOCCP)-mRNA-PPH, (IOCCP-2k PEI)-mRNA-PPH, (IOCCP-25k PEI)-mRNA-PPH and Lipofectamine 2000-mRNA at a concentration of 1 μg/mL mRNA for 24 h. The untreated cells’ viability was normalized to 100% for all cell lines. * *p* < 0.05, ns = statistically insignificant between each NP-mRNA-PPH/lipofectamine and the untreated group.

To further investigate the biocompatibility of the four NP-mRNA-PPH complexes, the cytotoxicity of NP-mRNA-PPHs was evaluated by assessing the viability of cells treated with the NP-mRNA-PPH complexes of 1 µg/mL mRNA using Alamar Blue assay (Figure 6). The viability of untreated cells was set as 100% and compared to the viability of cells treated with NP-mRNA-PPH complexes and Lipofectamine 2000. The viability of 4T1 cells treated with four different NP-mRNA-PPHs was comparable, ranging from 90% to 94%. In comparison, the viability of 4T1 cells treated with Lipofectamine 2000 was slightly higher at 96%. For HepG2, (IOCCP)-mRNA-PPH demonstrated excellent biocompatibility, with viability of 97% observed in cells treated with this formulation, while other NP-mRNA-PPH complexes and Lipofectamine 2000-mRNA resulted in lower values. The effects of (IOSPM)-mRNA-PPH, (IOCCP)-mRNA-PPH, and (IOCCP-2k PEI)-mRNA-PPH on C6 cell viability were similar, with viabilities of 92%, 86%, and 88%, respectively. (IOCCP-25k PEI)-mRNA-PPH demonstrated the lowest cell viability across all three cell lines among four NP-mRNA-PPH formulations. The lower biocompatibility observed with (IOCCP-25k PEI)-mRNA-PPH could be attributed to the high molecular weight of 25k PEI in the core material, which is often associated with increased cytotoxicity [32]. This high toxicity contributes to the reduced transfection efficiency (Figure 5). In summary, all NP-mRNA-PPH complexes, except for the (IOCCP-25k PEI)-mRNA-PPH complex, demonstrated excellent biocompatibility that is comparable to, if not greater than, that of Lipofectamine 2000-mRNA in the three cancer cell lines. 

## 4. Conclusions

Our studies of four NP-mRNA-PPH mRNA delivery platforms have led to the identification of a highly effective mRNA delivery platform, (IOCCP)-mRNA-PPH. This platform has demonstrated successful delivery of mRNA into multiple hard-to-transfect cancer cell lines in vitro. While all four NP-mRNA-PPH complexes showed similar profiles in terms of mRNA protection and cellular uptake, variations in surface chemistry and zeta potential of the core material resulted in differences in mRNA release behavior, biocompatibility, and ultimately, transfection performance. Notably, (IOCCP)-mRNA-PPH, which lacks a highly positively charged PEI on the core material surface, offers several advantages, including increased cellular internalization of nanoparticles, easy release of mRNA, and improved biocompatibility. Overall, our findings suggest that (IOCCP)-mRNA-PPH represents a highly promising mRNA delivery platform for the treatment of hard-to-transfect cancers.

## Data Availability

Data can be available upon request from the authors.

## Supplementary Materials

The following supporting information can be downloaded at: https://www.mdpi.com/article/10.3390/pharmaceutics15071946/s1, Figure S1: Cellular uptake and endosomal escape studies of mRNA-PPH on various cancer cell lines; Figure S2: FTIR spectra of (a) four nanoparticle cores modified with a charge-bearing thin layer and (b) the outer coating polymer component perfluoroheptanoic acid-polyethyleneimine (PFHA-PEI); Table S1: mRNA encapsulation efficiency and mRNA loading of nanocomplex; Figure S3: Size stability of NP-mRNA-PPHs in two cell media containing 10% serum (DMEM and RPMI); Figure S4: Confocal fluorescence images of 4T1, HepG2 and C6 cancer cell lines transfected with various NP-mRNA-PPH complexes; Figure S5: Size stability of the (IOCCP)-mRNA-PPH complexes under two storage conditions.
