# Supplementary material for: Iron Oxide Nanoparticle-Mediated mRNA Delivery to Hard-to-Transfect Cancer Cells
Source: Pharmaceutics. 2023 Jul 14;15(7):1946. doi: 10.3390/pharmaceutics15071946 (PMC10384052; doi:10.3390/pharmaceutics15071946)
Supplement: Supplementary file 1 [file pharmaceutics-15-01946-s001.zip › pharmaceutics-2453140-supplementary.pdf]

pharmaceutics

Supplementary Materials: Iron Oxide Nanoparticle-Mediated mRNA Delivery to Hard-to-Transfect Cancer Cells

Jianxi Huang, Guanyou Lin, Taylor Juenke, Steve Chung, Nicholas Lai, Tianxin Zhang, Tianyi Zhang and Miqin Zhang *

Department of Materials Science and Engineering, University of Washington, Seattle 98195, USA
* Correspondence: mzhang@uw.edu

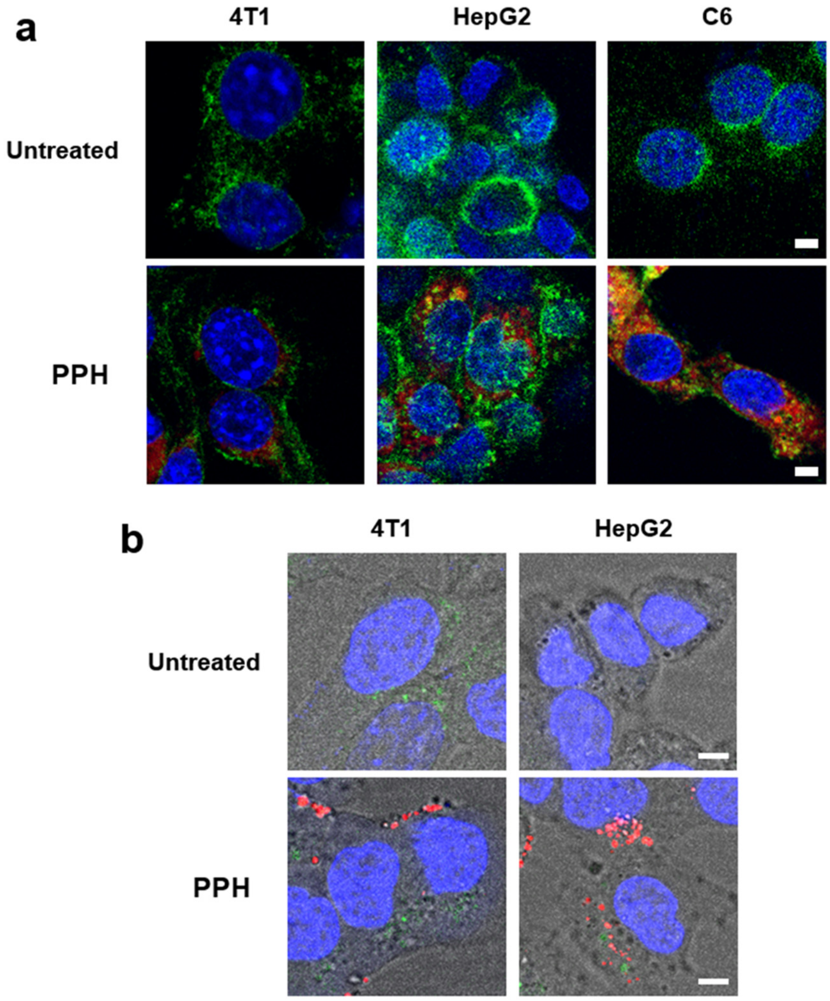

Figure S1. Cellular uptake and endosomal escape studies of mRNA-PPH on various cancer cell lines. (**a**) Confocal images of untreated cells (top row) and cells treated with mRNA-PPH (red, bottom row) showing that mRNA-PPH (red) was able to transport across cancer cell membranes (green). PPH in mRNA-PPH was labeled by Cy5 (red) before complexation into mRNA-PPH. mRNA-PPH was added to cell culture and incubated for 8 hours. DAPI nuclear was stained in blue. Scale bar is 5 µm. (**b**). Confocal images of untreated cells (top row) and cells treated with mRNA-PPH (bottom row) showing that the signal of mRNA-PPH (red) was distinguished from the signal of endosomes (green). These results indicated the mRNA was able to escape from endosomes, medicated by PPH. Cy5-labeled mRNA was complexed with PPH and added to cell cultures and incubated for 12 hours before adding Lysotracker Red reagent and incubated for another 1 hour. DAPI nuclear was stained in blue. Scale bar is 5 µm.

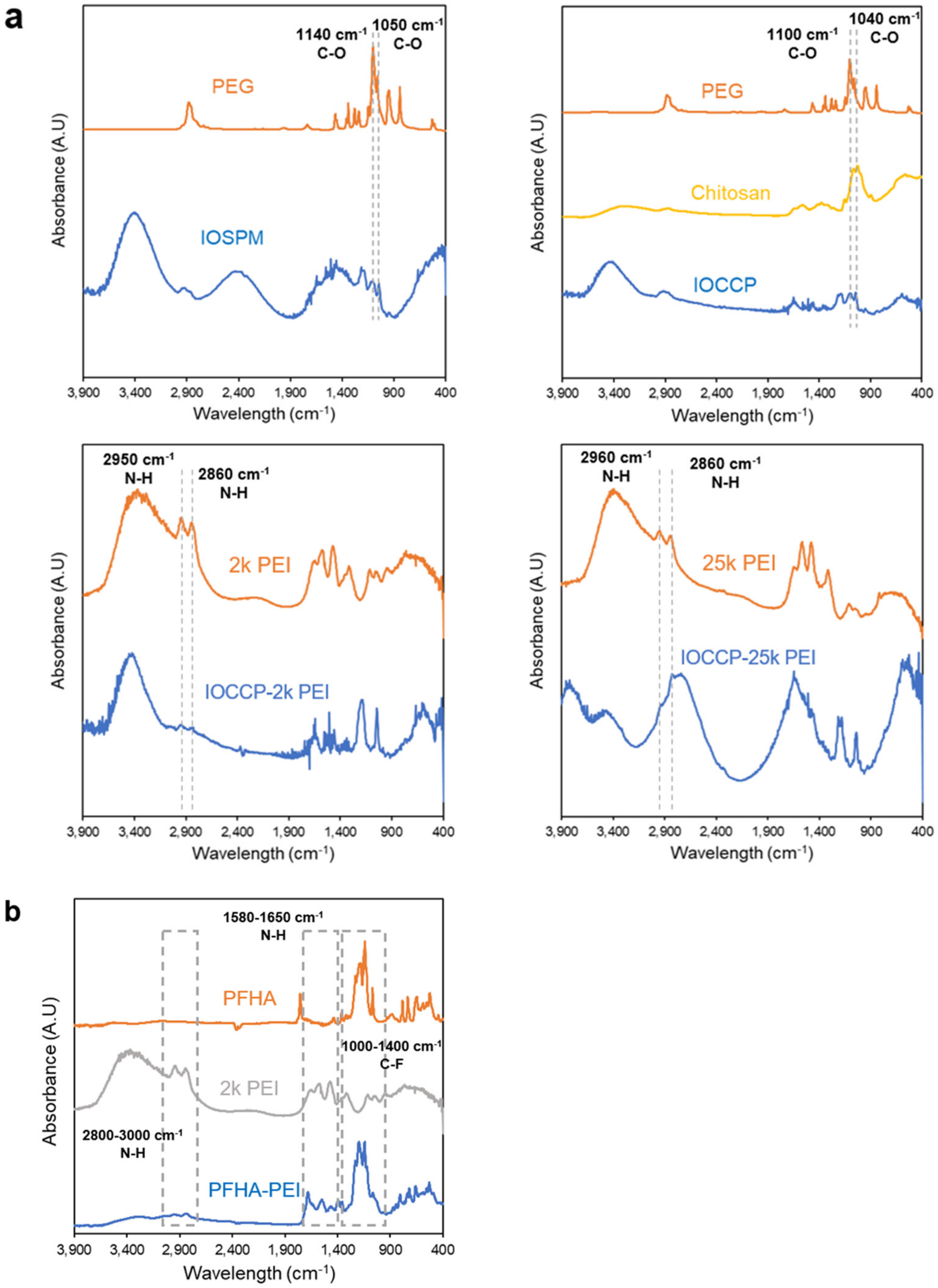

Figure S2. FTIR spectra of (**a**) four nanoparticle cores modified with a charge-bearing thin layer and (**b**) the outer coating polymer component perfluoroheptanoic acid-polyethyleneimine (PFHA-PEI). The IOSPM core has absorbance peaks of PEG at 1050–1140 cm^{-1}, which correspond to the stretching of C-O bonds. The IOCCP core inherits the peaks at around 1100 cm^{-1} and 1040 cm^{-1} from PEG's and chitosan's C-O stretching respectively. Two characteristic peaks of 2k PEI/25k PEI at 2810–2840 cm^{-1} and 2930–2970 cm^{-1} were found in both IOCCP-2k PEI and IOCCP-25k PEI, which correspond to N-H stretching in PEI. For the coating polymer component PFHA-PEI, the spectrum exhibited both characteristic peaks of C-F stretching from PFHA at 1000–1400 cm^{-1} and N-H bending at 1580–1650 cm^{-1}) and N-H stretching at 2800–3000 cm^{-1}) from PEI.

Table S1. mRNA encapsulation efficiency and mRNA loading of nanocomplex.

Material	mRNA Encapsulation Efficiency	mRNA loading [1]
(IOSPM)-mRNA-PPH	70.6% ± 12.0%	4.2% ± 0.7%
(IOCCP)-mRNA-PPH	83.7% ± 6.4%	4.9% ± 0.4%
(IOCCP-2k PEI)-mRNA-PPH	79.6% ± 22.7%	4.7% ± 1.4%
(IOCCP-25k PEI)-mRNA-PPH	82.6% ± 5.1%	4.9%± 0.3%

[1] mRNA loading (in weight percentage) was defined by the following equation, based on the measured mRNA encapsulation efficiency and the assumption that other components (NP, PPH) were fully encapsulated:

$$mRNA\ loading\ = \frac{mRNA\ mass * mRNA\ encapsulation\ efficiency}{Total\ complex\ mass} = \frac{mRNA\ mass * mRNA\ encapsulation\ efficiency}{NP\ mass +\ mRNA\ mass * mRNA\ encapsulation\ efficiency + PPH\ mass}$$

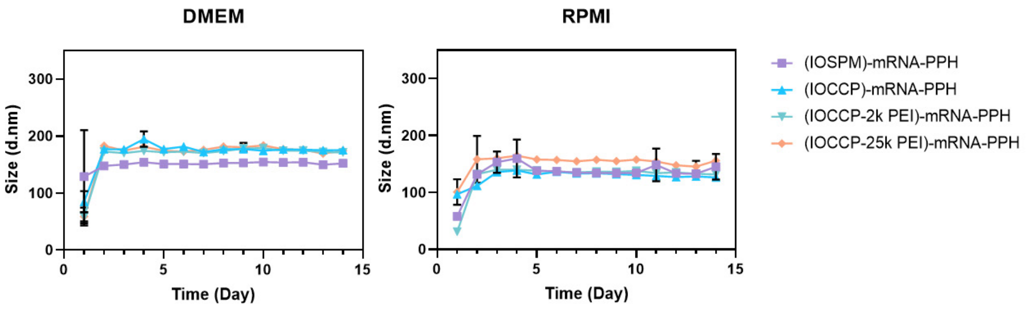

Figure S3. Size stability of NP-mRNA-PPHs in two cell media containing 10% serum (DMEM and RPMI). The hydrodynamic sizes are presented as z – average size.

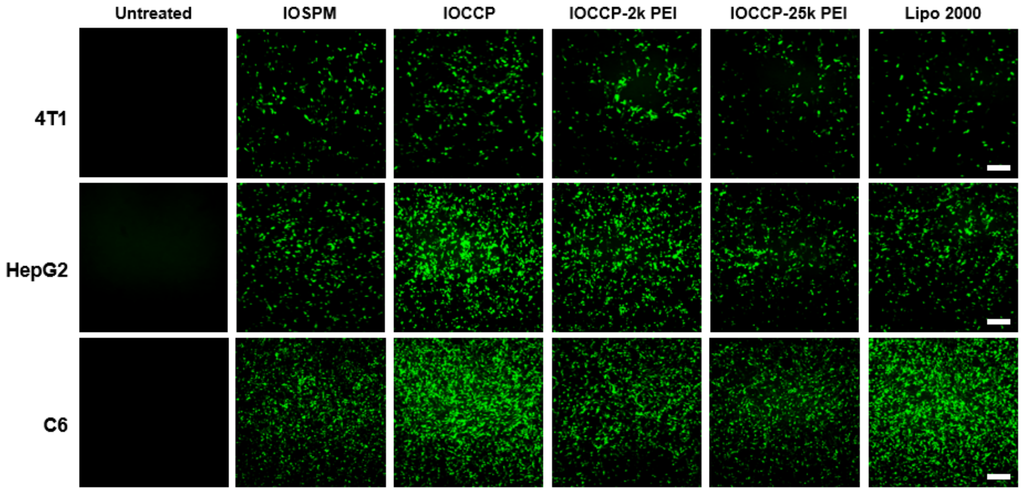

Figure S4. Confocal fluorescence images of 4T1, HepG2 and C6 cancer cell lines transfected with various NP-mRNA-PPH complexes (named according to their core materials (top row) and lipofectamine 2000 (Lipo 2000). Scale bar is 200 µm. The IOCCP-mRNA-PPH complex demonstrated the highest transfection efficiency in all three cancer cells, as compared to other NP-mRNA-PPH complexes.

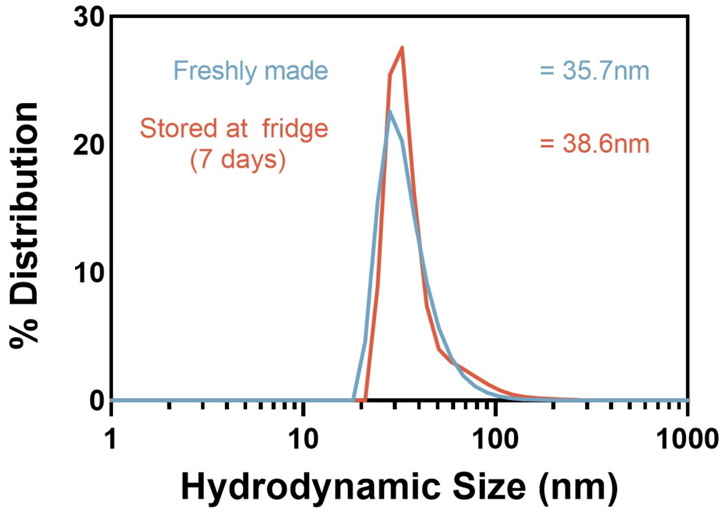

Figure S5. Size stability of the (IOCCP)-mRNA-PPH complexes under two storage conditions. The samples were freshly prepared within an hour (blue) and stored at 4 °C fridge for 7 days (red). The sample stored at fridge showed similar size distribution as that of the freshly made one.
